# Adherence to Drinking Guidelines and Reasons for Alcohol Consumption Cessation in the Southern Cone of Latin America – Findings from the CESCAS Study

**DOI:** 10.5334/gh.840

**Published:** 2021-01-04

**Authors:** Inge A. T. van de Luitgaarden, Pablo E. Gulayin, Laura Gutierrez, Matías Calandrelli, Nora Mores, Jacqueline Ponzo, Fernando Lanas, Ilse C. Schrieks, Diederick E. Grobbee, Joline W. J. Beulens, Vilma Irazola

**Affiliations:** 1 Instituto de Efectividad Clínica y Sanitaria, Buenos Aires, AR; 2 Julius Global Health, Julius Center for Health Sciences and Primary Care, University Medical Center Utrecht, Utrecht University, Utrecht, NL; 3 Julius Clinical, Zeist, NL; 4 Facultad de Ciencias Médicas, Universidad Nacional de la Plata, la Plata, AR; 5 Sanatorio San Carlos, Bariloche, Río Negro, AR; 6 Municipalidad de Marcos Paz, Buenos Aires, AR; 7 Facultad de Medicina, Universidad de la República, Montevideo, UY; 8 Universidad de La Frontera, CIGES, Temuco, CL; 9 Amsterdam University Medical Center, location VUmc, Amsterdam Cardiovascular Sciences research institute, Department of Epidemiology and Biostatistics, Amsterdam, NL

**Keywords:** alcohol consumption, adherence, guidelines, alcohol cessation, epidemiology

## Abstract

**Introduction:**

Alcohol consumption is a risk factor for morbidity and mortality globally. Consumption levels in Southern Latin America are among the highest in the world.

**Objectives:**

To describe consumption patterns and adherence to guidelines in the general adult population of Southern Latin America, as well as exploration of reasons for alcohol cessation and the advising role of the health worker in this decision.

**Methods:**

In 7,520 participants from the Centro de Excelencia en Salud Cardiovascular para el America del Sur (CESCAS) cohort, consumption patterns were described and the proportion excessive drinkers (i.e. >7 units/week for women and >14 for men or binge drinking: >4 (women) or >5 (men) units at a single occasion) was calculated. Former drinkers were asked if they had quit alcohol consumption on the advice of a health worker and/or because of health reasons. Furthermore, among former drinkers, multivariable logistic regression analysis was performed to assess which participant characteristics were independently associated with the chance of quitting consumption on a health worker’s advice.

**Results:**

Mean age was 54.8 years (SD = 10.8), 42% was male. Current drinking was reported by 44.6%, excessive drinking by 8.5% of the population. In former drinkers, 23% had quit alcohol consumption because of health reasons, half of them had additionally quit on the advice of a health worker. The majority of former drinkers however had other, unknown, reasons. When alcohol cessation was based on a health worker’s advice, sex, country of residence, educational status and frequency of visiting a physician were independent predictors.

**Conclusion:**

In this Southern American population-based sample, most participants adhered to the alcohol consumption guidelines. The advising role of the health worker in quitting alcohol consumption was only modest and the motivation for the majority of former drinkers remains unknown. A more detailed assessment of actual advice rates and exploration of additional reasons for alcohol cessation might be valuable for alcohol policy making.

## Introduction

Alcohol consumption accounts for 5.3% of all deaths and 5.1% of all disability-adjusted life years (DALYs) worldwide. These percentages are even slightly higher in the region of the Americas: 5.5% of all deaths and 6.7% of all DALYs are attributable to alcohol [1,2]. Nevertheless, alcohol consumption is culturally accepted as a social habit in society, which makes it challenging to effectively intervene on consumption patterns in the population. According to latest estimations, the annual alcohol consumption worldwide per person is 6.4 liters of pure alcohol [2]. With a mean annual alcohol consumption of 10 liters of pure alcohol per capita, alcohol consumption in the region of Southern Latin America (Chile, Argentina and Uruguay) is higher than in the neighbouring countries and also higher than in most other regions of the world, only exceeded by some European countries and similar to North America [2]. However, limited observational data on habitual alcohol consumption is available for Latin American countries [3].

Alcohol shows a dose-response relationship with a variety of diseases, such as some types of cancers, hemorrhagic stroke and liver disease [4]. By contrast, alcohol in moderation has been repeatedly suggested to have potential beneficial effects on ischemic cardiovascular disease and type 2 diabetes mellitus [5,6]. Recommendations on consumption limits have been adjusted over the years. In Argentina and Uruguay, the current advice is to limit alcohol consumption to no more than 14 units/week for men and 7 units/week for women [7,8], similar to Europe and the United States [9,10]. Chile follows the guideline of the World Health Organization, which is similar to the one above [11].

At the same time, surveys among physicians in Argentina and Uruguay showed that physicians were not satisfied with their knowledge on consumption guidelines [12,13]. Health workers, however, play an important role in counselling people on lifestyle modifications, as part of the chronic care model [14,15]. This healthcare strategy provides a patient-centred approach in which the focus is on prevention, patient education and self-management of disease and risk factors [14]. Advice on responsible alcohol consumption is part of the program. It has been suggested that certain patient characteristics (such as male sex, being young or middle-aged, low education and presence of obesity) are associated with a higher chance of getting advice on alcohol consumption [16], evidence is however limited.

In the present study we described, based on the present alcohol consumption patterns, adherence to consumption guidelines in the general population of Southern Latin America. This extends the work that was previously done on epidemiology of alcohol consumption in this population [17]. Moreover, we examined reasons for quitting consumption of alcohol among former drinkers and the advising role of the health worker in this decision. Lastly, we investigated which population characteristics were associated with alcohol consumption cessation on the advice of a health worker and which were associated with quitting because of health reasons.

## Methods

### Study population

The Centro de Excelencia en Salud Cardiovascular para el America del Sur (CESCAS) I study is a population-based study with multistage probabilistic sampling from four representative mid-sized cities in the Southern Cone of Latin America, aimed to examine CVD and its risk factors. Design and sampling method have been described elsewhere [18]. In short, 7,524 non-institutionalised participants between the age of 35 and 74 years, representing the general adult population in Argentina, Chile and Uruguay, were recruited. Recruitment took place between February 2010 and December 2012 from four cities: two in Argentina (Bariloche and Marcos Paz), one in Chile (Temuco) and one in Uruguay (Pando-Barros Blancos). All potential participants were invited through a letter of the site institution. The overall response rate was 73.4%, which was similar for men and women and for each of the four locations. The study was carried out following the guidelines for the protection of the rights of human volunteers and complies with the Declaration of Helsinki. The study protocol has been approved by Institutional Review Boards (IRBs) for all participating institutions in Argentina, Chile, Uruguay and USA. All study participants signed written informed consent. In the present study, participants without data on alcohol consumption (N = 4) were excluded.

### Assessment of alcohol consumption

Alcohol consumption was self-reported by a beverage-specific quantity-frequency structured questionnaire. Participants were asked to report their habitual weekly consumption of units of beer, wine and spirits and whether they had been binge drinking in the past month. Binge drinking is defined as the consumption of more than five units at one occasion (i.e. within approximately two hours) for men and more than four units for women [9]. A standard drink in the Americas in general contains approximately 14 grams of alcohol. If less than one standard unit of alcohol was consumed during the week, alcohol consumption was recorded as zero. Moreover, participants who did not currently consume alcohol were asked whether they were lifetime abstainers or former drinkers (former drinking defined as stopped drinking at least one year ago). Former drinkers were asked two questions about their motivation for quitting consumption: whether they had stopped drinking for health reasons (yes/no), and whether they had stopped drinking on the advice of a health worker (yes/no). Alcohol consumption was categorized into sex-specific consumption categories. For men the following categories were adopted: abstention, former drinker, current drinker (light [< 1 units/week], moderate [1–14 units/week], heavy drinkers [> 14 units/week] and binge drinkers [> 5 units at one occasion (~2 hours), more than once a month]). Women were categorized into: abstention, former drinker, current drinker ([light < 1 unit/week], moderate [1–7 units/week], heavy [> 7 units/week] and binge drinkers [> 4 units at one occasion (~2 hours), more than once a month]). All participants that indicated binge drinking in the previous month were categorized as binge drinkers, regardless of their habitual alcohol consumption pattern. Heavy drinking and binge drinking were both seen as excessive drinking patterns, since these levels of consumption do not comply with the guidelines. These participants were thus categorized as excessive drinkers.

### Covariates

Study data were collected during a home visit and a clinical visit. Information on personal and demographic details was obtained during the home visit. Age was divided into four equal age categories (35–44, 45–54, 55–64 and 65–74 years).

Education level was self-reported based on highest ascertainment and stratified according to three categories: 1) low (primary education completed), 2) intermediate (secondary education completed) and 3) high (university/college completed). Smoking status was divided into 1) never smoking, 2) former smoking and 3) current smoking. Physical activity level was self-reported and converted into metabolic equivalents (METs). Low physical activity was defined as a METs score < 600 MET-minutes per week, normal/high physical activity as a METs score ≥ 600 MET-minutes [19].

Based on self-reported chronic disease history and presence of cardiometabolic risk factors, the following division was made: 1) no history of disease, 2) no history of disease but high CVD risk (based on Framingham risk score: >20% 10 year risk [20]), 3) history of cardiovascular disease (including angina, myocardial infarction, stroke, transient ischemic attack [TIA], aortic aneurysm, peripheral artery disease, heart failure and procedures related to CVD), 4) malignancy (including all types of cancer), 5) respiratory disease (including asthma, chronic bronchitis, COPD and history of tuberculosis) and 6) multimorbidity (including at least two disease categories: cardiovascular disease, malignancy and/or respiratory disease).

Access to healthcare was self-reported by questionnaire, including the following determinants: having a primary physician (yes/no), ever needed healthcare but could not (yes/no), number of visits to a healthcare worker in the past year: 1) less than twice a year, 2) between 2 and 12 times a year and 3) more than 12 times a year, and consultation of alternative medicine in the past year (yes/no).

Hypertension was defined as mean systolic blood pressure ≥140 mmHg and/or mean diastolic blood pressure ≥90 mmHg and/or current use of antihypertensive medication. Dyslipidaemia was present if at least one of the lipid levels was elevated: total cholesterol ≥240 mg/dl, an LDL-cholesterol ≥160 mg/dl, an HDL-cholesterol <40 mg/dl and/or a triglyceride level ≥200 mg/dl, or if lipid-lowering medication was used. Type 2 diabetes mellitus (T2DM) was defined as fasting glucose ≥126 mg/dl and/or self-reported history of diabetes and/or current use of insulin or antidiabetic medication.

Physical measurements were performed during the clinical visit by trained staff. Blood pressure was measured three times using a standard mercury or aneroid sphygmomanometer, and the mean was used for the analyses. Height and weight were measured twice in light indoor clothes without shoes and the average was used for further analyses. Body-mass index (BMI) was calculated as measured weight in kilograms divided by the square of height in meters. Obesity was defined as a BMI ≥ 30kg/m2. Overnight fasting blood samples were drawn for measurement of lipids, creatinine and glucose.

### Statistical analysis

All statistical analyses were performed using IBM SPSS 26.0 for Windows and R studio version 3.6.1. Estimates were weighed to represent the general adult population aged 35–74 years in the study sites, according to the study sampling design. We tabulated demographic and lifestyle factors, as well as history of disease and comorbidities, stratified by alcohol consumption category. To evaluate the validity of self-reported alcohol intake, we performed one-way ANOVA with linear polynomial contrast between alcohol consumption categories and HDL-c levels, since HDL-c is known to be an objective biomarker of alcohol consumption, even at moderate levels of consumption [21]. Additionally, for each alcohol consumption category, we calculated the median alcohol consumption in grams per week. We furthermore calculated the proportion of participants that did not adhere to the drinking guidelines (i.e. excessive drinkers: heavy drinkers and binge drinkers). We graphically presented the distribution of never, former, current and excessive drinkers stratified by sex and disease category.

To further explore reasons for alcohol consumption cessation in former drinkers across different disease categories, we calculated the proportion of former drinkers that indicated to have quit because of health reasons, as well as the proportion that quit because they were advised to do so, stratified by sex and stratified by disease category. We additionally calculated the proportion that quit alcohol consumption because they were advised to do so *and* because of health reasons, the proportion that quit because of health reasons but was not advised by a health worker, and finally the proportion that indicated not to have quit for any of the two reasons.

Furthermore, among the former drinkers, we examined what kind of participants more often quit because they were advised to do so, by analyzing which determinants independently contributed to the chance of having quit alcohol consumption on a health worker’s advice. We performed multivariable logistic regression analyses with ‘quit because of being advised’ as the outcome variable. The number of ‘events’ (i.e. the number of participants that quit because they were advised) was 194, which allowed for a maximum of 19 independent predictors according to the events per variable (EPV) 1–10 rule of thumb, that states that for each candidate predictor at least 10 outcome events are required to warrant reliable modelling [22]. We included sociodemographic determinants (age, sex, country of residence, education level, smoking status, obesity), disease determinants (history of disease, using the previously defined categories) and determinants of access to healthcare (having a primary physician, number of visits to a physician in the past year, consultation of alternative medicine and inability to access healthcare). We performed backward selection of determinants, based on the Akaike Information Criterion (AIC), to obtain the predictors that were independently related to the outcome. We performed the same analyses with health reasons as rationale for alcohol consumption cessation as outcome with 348 events.

## Results

Mean age was 54.8 years (SD = 10.8), 42% of the participants was male. Alcohol consumption categories were positively associated with HDL-c levels (P for trend < 0.001). Therefore, the self-reported alcohol intake assessment was regarded as a valid measure. Current drinking was reported by 44.6%. The percentage excessive drinkers (i.e. not adherent to the drinking guidelines) was 8.5% of the total population. Differences in consumption patterns were more pronounced for sex than for disease category. Men (59%) were more often current drinkers than women (32%) and the proportion excessive drinkers was also higher in men (13.9%) than in women (3.7%), regardless of disease category (Supplementary Figure 1).

When considering the current drinkers only, the most common consumption pattern was moderate alcohol consumption (54.4%). However, 19% was considered an excessive drinker, and this percentage was higher in men (23.6%) than in women (11.5%). Male excessive drinkers also consumed more alcohol than their female counterparts (median alcohol consumption: 139 g/week [25–75 percentile: 52–265] in men versus 101 g/week [25–75 percentile: 52–138] in women). Wine was the most consumed type of beverage in both men (54%) and women (51%), however in binge drinkers the most common beverage type was beer (43% in men and 42% in women). Excessive drinkers were often younger and were more often current smokers (Tables 1 and 2).

**Table 1:** Participants characteristics of 4359 female CESCAS participants.

	N	In line with guidelines				Excessive drinkers	

		Lifetime abstainers	Former drinkers	< 1 unit/week	1–7 units/week	>7 units/week	Binge drinkers

**% of participants**	*4359*	48.5	19.6	12.1	16.1	2.0	1.7
**Age group**							
35–44 years	*1009*	44.3	18.4	14.2	18.5	2.0	2.6
45–54 years	*1240*	48.2	19.3	11.9	17.3	2.0	1.4
55–64 years	*1180*	52.8	20.9	11.4	11.9	1.9	1.1
65–74 years	*930*	54.3	21.7	7.8	13.5	2.3	0.4
**Country**							
Argentina	*2400*	53.2	11.9	13.6	15.9	3.8	1.7
Chile	*1027*	49.3	21.9	9.9	16.6	0.7	1.6
Uruguay	*932*	34.9	31.0	15.4	15.2	1.7	1.8
**Obesity (BMI > 30)**	*1845*	52.4	18.7	12.0	13.8	1.4	1.6
**Smoking status**							
Current smoking	*1042*	35.0	18.8	14.9	23.9	3.7	3.7
Former smoking	*878*	39.6	22.6	13.1	20.4	2.0	2.3
Never smoking	*2424*	58.7	18.8	10.4	10.5	1.2	0.4
**Education level**							
Primary education	*2028*	54.9	19.9	10.8	11.5	1.5	1.4
Secondary education	*1590*	49.7	19.7	11.5	15.6	1.8	1.6
University	*736*	36.7	19.0	15.0	24.0	3.2	2.1
**Low physical activity**	*1661*	50.3	19.7	11.5	15.0	1.8	1.8
**Presence or history of disease**							
No history of disease	*2621*	46.9	18.4	13.4	17.4	1.9	1.9
High CVD risk	*358*	54.3	23.5	7.9	10.5	1.5	2.4
Cardiovascular disease	*490*	49.0	25.2	10.2	11.3	2.0	2.3
Respiratory disease	*489*	46.8	20.3	10.9	18.8	2.9	0.4
Malignancy	*140*	47.0	22.3	9.6	18.0	2.2	0.9
Multimorbidity	*261*	64.6	17.1	7.9	8.0	1.4	1.0
**Comorbidities**							
Diabetes	*646*	53.5	21.3	11.5	11.2	1.0	1.4
Hypertension	*1956*	53.0	21.9	9.5	12.3	1.7	1.5
Dyslipidemia	*2219*	49.9	21.3	11.3	14.5	1.9	1.1
**Total cholesterol (mg/dl)**		201 ± 42	201 ± 44	203 ± 41	203 ± 41	206 ± 37	199 ± 40
**HDL cholesterol (mg/dl)**		48 ± 12	48 ± 13	49 ± 12	51 ± 13	52 ± 14	50 ± 12
**Median alcohol consumption in g/week**		0 [0, 0]	0 [0, 0]	0 [0, 0]	28 [14, 46]	124 [101, 155]	41 [14, 83]

Values represent percentages; *absolute numbers*; means ± standard deviations; medians [25–75 percentiles]. Abbreviations: BMI (body mass index), CESCAS (Centro de Excelencia en Salud Cardiovascular para el Cono Sur), CVD (cardiovascular disease), HDL (high-density cholesterol).

**Table 2:** Participant characteristics of 3161 male CESCAS participants.

	N	In line with guidelines				Excessive drinkers	

		Lifetime abstainers	Former drinkers	<1 unit/week	1–14 units/week	>14 units/week	Binge drinkers

**% of participants**	*3161*	24.4	16.8	11.5	33.5	4.0	9.9
**Age group**							
35–44 years	*705*	27.1	13.1	12.2	32.4	2.7	12.6
45–54 years	*831*	23.2	16.3	11.5	34.3	3.7	11.1
55–64 years	*935*	20.8	20.9	11.6	34.3	5.9	6.4
65–74 years	*690*	24.5	23.0	8.7	34.0	5.3	4.5
**Country**							
Argentina	*2400*	25.6	12.2	13.9	35.8	5.9	6.6
Chile	*1027*	27.7	18.4	10.0	29.4	1.2	13.3
Uruguay	*932*	11.7	22.9	10.0	39.9	7.5	7.9
**Obesity (BMI > 30)**	*1067*	23.6	16.4	12.0	34.6	3.5	9.9
**Smoking status**							
Current smoking	*960*	18.1	12.8	12.0	35.6	5.8	15.7
Former smoking	*1103*	19.0	22.2	12.5	33.7	4.6	8.0
Never smoking	*1076*	35.4	15.4	10.2	31.1	1.7	6.3
**Education level**							
Primary education	*1380*	22.1	17.9	11.9	31.9	5.0	11.2
Secondary education	*1200*	24.7	17.6	11.3	33.1	3.5	9.8
University	*579*	26.8	13.8	11.3	36.4	3.2	8.5
**Low physical activity**	*892*	28.7	17.5	11.2	31.3	3.8	7.5
**Presence or history of disease**:							
No history of disease	*1398*	25.7	14.6	12.0	34.2	3.0	10.4
High CVD risk	*852*	22.3	15.7	13.6	36.3	5.7	6.5
Cardiovascular disease	*452*	21.9	25.9	8.2	30.3	4.9	8.7
Respiratory disease	*292*	24.5	17.5	9.9	27.6	4.2	16.3
Malignancy	*55*	26.7	25.3	8.9	32.8	4.5	1.9
Multimorbidity	*111*	19.5	23.8	5.8	34.9	6.8	9.2
**Comorbidities**							
Diabetes	*414*	23.1	23.5	12.2	30.0	3.4	7.7
Hypertension	*1625*	21.4	17.7	11.1	34.5	5.5	9.9
Dyslipidemia	*2098*	25.2	17.0	12.4	32.8	3.2	9.4
**Total cholesterol (mg/dl)**		197 ± 39	192 ± 44	204 ± 42	206 ± 42	209 ± 45	207 ± 38
**HDL cholesterol (mg/dl)**		40 ± 11	41 ± 12	40 ± 9	43 ± 11	48 ± 18	46 ± 16
**Median alcohol consumption in g/week**		0 [0, 0]	0 [0, 0]	0 [0, 0]	51 [26, 86]	264 [210, 331]	83 [39, 160]

Values represent percentages; *absolute numbers*; means ± standard deviations; medians [25–75 percentiles]. Abbreviations: BMI (body mass index), CESCAS (Centro de Excelencia en Salud Cardiovascular para el Cono Sur), CVD (cardiovascular disease), HDL (high-density cholesterol).

## Reasons for quitting consumption

**Figure 1:**
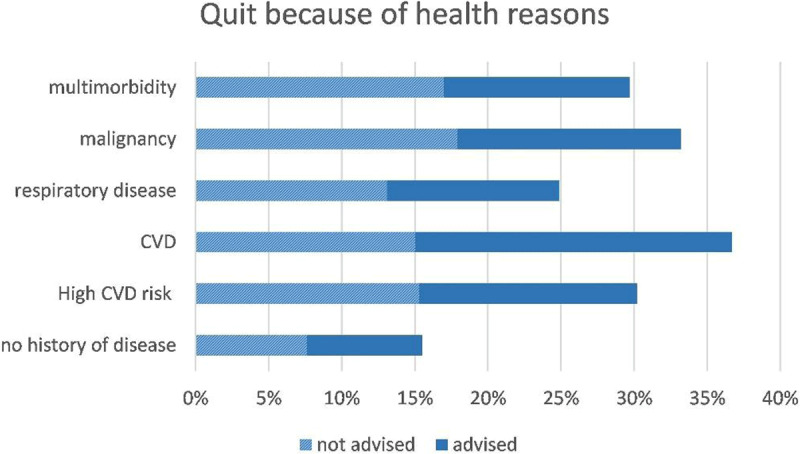
Percentages of CESCAS participants that quit because of health reasons, per disease category, with proportions of them being additionally advised of not advised.

**Figure 2:**
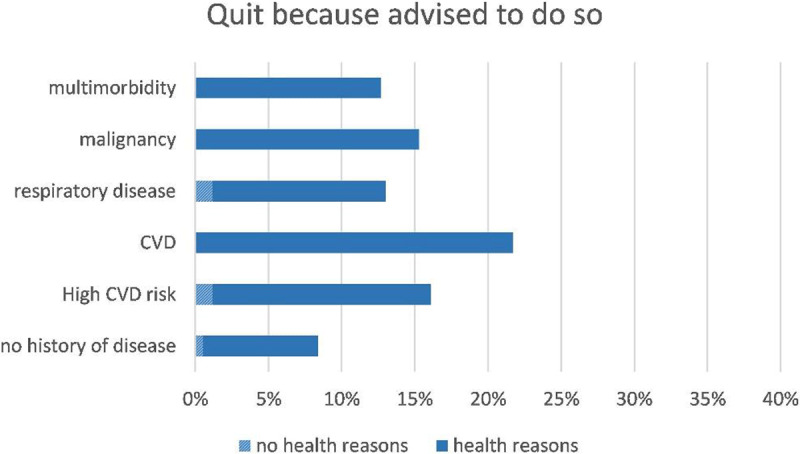
Percentages of CESCAS participants that quit because they were advised to do so, per disease category, with proportions of them that additionally quit because of health reasons.

The total number of former drinkers in this cohort was 1334, 1263 (95%) of them provided information on whether their health and/or advice by a health worker were reasons for alcohol cessation: 23% indicated to have quit alcohol consumption because of health reasons and 12% because they were advised by a health worker. Men more often quit because of health reasons than women (27% vs 19%) and also more often after advice of a health worker (17% versus 8%) (Table 3). The percentages also differed per disease category: in general, participants with a history of disease more often quit because of health reasons or because of advice than participants without a history of disease. Participants with cardiovascular disease most often indicated to have quit alcohol consumption because of advice and/or health reasons (Figures 1 and 2). Almost all participants who were advised by a health worker indicated to have quit because of health reasons as well. However, of all participants that quit because of health reasons, only approximately half of them was also advised by a health worker (Figures 1 and 2). The majority of former drinkers (77%) answered ‘no’ to both questions and thus had other reasons for quitting alcohol consumption than health or advice, their motivation remains unknown.

**Table 3:** Motivation for alcohol consumption cessation in 1263 former drinkers from the CESCAS cohort.

		Quit Because of Health Reasons			

			Yes	No	Total

Quit Because of A Health Worker’s Advice	All (N = 1263)	**Yes**	11.6%	0.5%	12.1%
		**No**	11.0%	76.9%	87.9%
		**Total**	22.5%	77.5%	100%
	Women (N = 731)	**Yes**	7.9%	0.3%	8.2%
		**No**	10.9%	80.9%	91.8%
		**Total**	18.8%	81.2%	100%
	Men (N = 532)	**Yes**	16.3%	0.9%	17.2%
		**No**	11.1%	71.7%	82.8%
		**Total**	27.4%	72.6%	100%

Participant characteristics that were independently associated with quitting consumption on a health worker’s advice are displayed in Table 4. Male sex and more than two visits to a physician in the past year were positively associated with alcohol consumption cessation on a health worker’s advice. High education (versus low education) and Chile as country of residence (versus Argentina) were negatively associated with this outcome. Similar associations were found for quitting because of health reasons, with the exception that country of residence was not an independent predictor for this outcome. Additionally, higher age, history of cardiovascular disease and malignancy were found to be independent predictors for alcohol cessation because of health reasons.

**Table 4:** Determinants of alcohol consumption cessation because of a health worker’s advice and because of health reasons in 1263 former drinkers.

Determinant	Because of advised to quit (Yes: N = 193) OR (95% CI)	Because of health reasons (Yes: N = 348) OR (95%)

Demographic determinants		

Male sex	**2.77 (1.79–4.30)**	**1.85 (1.30–2.65)**
Age	–	**1.02 (1.00–1.04)**
*Country (ref Argentina)*		
Chile	**0.53 (0.30–0.93)**	0.68 (0.43**–**1.06)
Uruguay	0.83 (0.55**–**1.27)	1.13 (0.79**–**1.61)
*Education (ref low education)*		
Intermediate education	1.89 (0.58**–**1.38)	0.98 (0.67**–**1.42)
High education	**0.37 (0.18–0.79)**	**0.57 (0.33–0.98)**
Disease determinants		

*History of disease: (ref no history of disease)*		
High CVD risk	–	1.34 (0.85–2.15)
Cardiovascular disease	–	**1.86 (1.18**–**2.93)**
Respiratory disease	–	1.35 (0.76–2.40)
Malignancy	–	**2.82 (1.04**–**7.63)**
Multimorbidity	–	0.90 (0.46–1.74)
Access to healthcare		

Having a primary physician	1.51 (0.96–2.38)	1.30 (0.89–1.89)
*Visits to a health professional (ref = 0–2/year)*		
2–12 per year	**1.95 (1.07**–**2.22)**	**1.48 (1.01**–**2.14)**
> 12 per year	**2.69 (1.03–4.05)**	**2.61 (1.38–4.93)**

Abbreviations: CI (confidence interval), CVD (cardiovascular disease), OR (odds ratio). Numbers in bold are associations that were found statistically significant (P < 0.05). The presented odds ratios are the risk estimates from the multivariable regression analyses after backward selection was performed.

## Discussion

In this general population cohort in the Southern Cone of Latin America, 59% of the males and 32% of the female reported current alcohol consumption. The proportion that did not adhere to the guidelines (i.e. both heavy drinkers and binge drinkers) was 8.5%, which was higher in men (13.9%) than in women (3.7%). Of the former drinkers, 23% indicated health reasons as motivation for alcohol consumption cessation, and only 12% indicated being advised by a health worker as reason. Men more often quit because of a health worker’s advice or health reasons than women. In about 50% the health worker’s advice played an additional role in quitting because of health reasons. Participants with a history of disease, in particular cardiovascular disease, more often quit because of advice and/or health reasons as compared to participants without a history of disease. However, the vast majority of former drinkers answered negative to both questions and apparently had other reasons for quitting. Sex, country of residence, educational status and frequency of visiting a physician were independent predictors of alcohol consumption cessation after advice of a health worker.

### Strengths and limitations

This is the first study to assess adherence to guidelines in the Southern Cone of Latin America in a large population-based sample. The multistage sampling method that was used to select the study population assured representativeness of the general adult population in four cities of Southern Latin America. The comprehensive data on alcohol consumption permitted us to compare consumption patterns with national guideline recommendations. Furthermore, we were able to evaluate the advising role of the health worker in alcohol consumption cessation and explored what kind of participants more often quit consumption because they were advised to do so.

Our study also has some important limitations. Firstly, information about the recommendations that were given by the health worker was only available for former drinkers. Therefore, the group that was advised to quit is only represented by the participants that were advised and subsequently quitted alcohol consumption. In reality, there must have been more participants that were advised to quit consumption, but decided not to. Likewise, there might have been participants who have been advised to only reduce alcohol consumption within the low-risk consumption limits. The proportion of participants that were advised on alcohol consumption limits is therefore likely to be underestimated in our study.

Moreover, since this is a general population cohort, and therefore a relatively healthy cohort, some of the disease categories were rather small. As a consequence, estimates in these categories might have been less precise. Finally, as in all epidemiological studies on alcohol consumption, the subjective nature of self-reported alcohol consumption, although in this study validated against an objective marker, might have led to an underestimation of the actual consumption [23].

### Comparison with other surveys

According to National Risk Factor Surveys that have been conducted in the three countries, men were more often current drinkers than women (range current drinking in men: 55–64% and current drinking in women: 37–43%) [11,24,25]. This is consistent with our population-based sample, except for the fact that current drinking rates in women were slightly lower (32%). Current drinking rates in the Southern Cone as estimated by the World Health Organization were higher with approximately 80% in men and 56% in women [2]. The Argentinean survey [24] used the same definition of excessive drinking as in our study and found that 26% of the current drinkers were excessive drinkers, whereas in our population 19% of the current drinkers were categorized as such. It is important to acknowledge that all these surveys have been held in general populations with age ranges from 15–64 years, while in our study age ranged between 35 and 74. Consumption of alcohol varies with age throughout the life course, particularly prevalent at younger age and decreasing at higher age, which might explain the differences we found [26].

### Determinants of alcohol consumption cessation

Our analysis showed a couple of factors that were associated with a higher chance of quitting alcohol consumption on a health worker’s advice, among people that already quit drinking. Male sex was one of the strongest predictors. Similar European research on adherence to alcohol consumption guidelines also showed that men were more often advised than women [27]. This is probably explained by the fact that men are more often excessive drinkers and therefore are advised more often. The number of visits to a physician might be a proxy for general health status, but clearly also the amount of time spent at a physician’s office might increase the chance of getting advice on alcohol consumption. The chronic care model also stresses the importance of adequate follow up visits [14]. Furthermore, low education, as a proxy for lower social economic status (SES), was positively associated with quitting alcohol consumption because of advice or health reasons. Although people with high SES often consume more alcohol than those with lower SES, previous research showed that alcohol consumption cessation in general occurs more often in low SES [28,29,30,31]. Possible explanations include the fact that those of a lower SES tend to experience more alcohol-related problems, regardless of level of consumption [32,33], and the higher rate of comorbidities in people with a lower SES as compared to those with a higher SES [34,35]. Both characteristics could lead to a higher advice rate in people with a low SES.

The difference in advice rates between countries, particularly between Chile and Argentina/Uruguay is interesting. Compared to Argentineans from the cities of Marcos Paz and Bariloche, former drinkers from Temuco in Chile had 50% lower odds of having quit because they were advised. We hypothesize that this might stem from national differences with respect to alcohol consumption limits. Until recently, consumption guidelines in Chile were clinically more flexible and dictated that up to three standard drinks of 14 g a day for women and up to four a day for men were regarded as within safe drinking limits, when limiting consumption to a few days per week [36]. This made Chile the country with the highest low-risk drinking limits in a study that compared alcohol consumption guidelines in 37 countries worldwide [37]. Attitudes and beliefs of participants as well as health workers towards low-risk consumption limits might differ between countries and therefore might be a potential explanation of the differences we found.

Interestingly, the majority of former drinkers indicated to have quit because of other reasons than health reasons or medical advice, even in the disease categories. Several US studies have proposed determinants such as simultaneous smoking cessation and certain sociodemographic factors to be positively associated with alcohol consumption cessation [28,30,31]. In a recent study in an Asian population, participants were asked about reasons for cessation: financial reasons, and to a lesser extent concerns about future health problems, were triggers to quit alcohol consumption [38]. Britton et al additionally provided ‘less social occasions to drink alcohol’ as reason for alcohol consumption cessation [29]. It would be valuable to further explore reasons for alcohol consumption cessation in our population, as public health initiatives to reduce alcohol consumption could possibly be tailored to this particular information.

## Conclusion

In conclusion, in this general population cohort in the Southern Cone of Latin America, most participants currently adhered to the alcohol consumption guidelines. However, in current drinkers almost one out of five was considered an excessive drinker. Current drinking and excessive drinking rates differed for sex and disease status. Health reasons were the motivation for alcohol consumption cessation for one–third of the former drinkers. The advising role of the health worker in quitting alcohol consumption was only modest in this population of former drinkers. The motivation for alcohol cessation for the majority of former drinkers remains unknown. Alcohol policy making could benefit from more information about reasons for alcohol consumption cessation in this population. Moreover, future research should be aimed at providing a more detailed overview of the current advice rates given by health workers to identify other elements for improvement in the regulation of alcohol consumption, which will contribute to a more adequate control of the burden of non-communicable diseases.

## Data Availability Statement

The data that support the findings of this study are available from the corresponding author, upon reasonable request.

## Additional File

The additional file for this article can be found as follows:

10.5334/gh.840.s1 Supplementary Figure 1. Alcohol consumption patterns per disease category in 3161 male and 4359 female CESCAS participants.
